# Hypophosphatasia

**DOI:** 10.3390/jcm10235676

**Published:** 2021-12-01

**Authors:** Symeon Tournis, Maria P. Yavropoulou, Stergios A. Polyzos, Artemis Doulgeraki

**Affiliations:** 1 Laboratory for the Research of Musculoskeletal System “Th. Garofalidis”, School of Medicine, National and Kapodistrian University of Athens, KAT General Hospital, 14561 Athens, Greece; 2 Endocrinology Unit, 1st Department of Propaedeutic Internal Medicine, National and Kapodistrian University of Athens, School of Medicine, Laikon General Hospital, 14561 Athens, Greece; myavropoulou@med.uoa.gr; 3 First Laboratory of Pharmacology, School of Medicine, Aristotle University of Thessaloniki, 54124 Thessaloniki, Greece; spolyzos@auth.gr; 4 Department of Bone and Mineral Metabolism, Institute of Child Health, 14561 Athens, Greece; doulgeraki@yahoo.com

**Keywords:** bone, alkaline phosphatase, arthropathy, asfotase alpha, fractures, tooth loss

## Abstract

Hypophosphatasia (HPP) is an inherited metabolic disease caused by loss-of-function mutations in the tissue non-specific alkaline phosphatase (*TNAP*) gene. Reduced activity of TNAP leads to the accumulation of its substrates, mainly inorganic pyrophosphate and pyridoxal-5′-phosphate, metabolic aberrations that largely explain the musculoskeletal and systemic features of the disease. More than 400 *ALPL* mutations, mostly missense, are reported to date, transmitted by either autosomal dominant or recessive mode. Severe disease is rare, with incidence ranging from 1:100,000 to 1:300,000 live births, while the estimated prevalence of the less severe adult form is estimated to be between 1:3100 to 1:508, in different countries in Europe. Presentation largely varies, ranging from death in utero to asymptomatic adults. In infants and children, clinical features include skeletal, respiratory and neurologic complications, while recurrent, poorly healing fractures, muscle weakness and arthropathy are common in adults. Persistently low serum alkaline phosphatase is the cardinal biochemical feature of the disease. Management requires a dedicated multidisciplinary team. In mild cases, treatment is usually symptomatic. Severe cases, with life-threating or debilitating complications, can be successfully treated with enzyme replacement therapy with asfotase alfa.

## 1. Introduction

Hypophosphatasia (HPP) is an inherited metabolic disease, characterized by low activity of tissue non-specific alkaline phosphatase (TNAP) due to mutations at the *ALPL* gene [1]. Low activity of TNAP leads to systematic accumulation of its substrates, namely inorganic pyrophosphate (PPi), a strong inhibitor of mineralization, and pyridoxal-5′-phosphate (PLP), a cofactor for several enzymes, which largely explain the musculoskeletal and systemic features of the disease. HPP is characterized by a wide spectrum of manifestations and severity, ranging from death in utero to dental complications only in children and adults, or asymptomatic carriers of *ALPL* mutations. There are more than 400 *ALPL* gene mutations (mostly missense) reported to date https://alplmutationdatabase.jku.at/portal/ (accessed on 27 November 2021), inherited by either autosomal dominant or recessive mode. Fortunately, severe disease is rare, with incidence ranging from 1:100,000 live births in Canada [2] to 1:300,000 in Europe [3], while the estimated prevalence of the less severe adult form is between 1:3100 to 1:508 in Europeans [3,4,5]. Since the first description of the disease by J.C. Rathbun in 1948 [6], there has been substantial progress in our knowledge on HPP, including the epidemiology, pathophysiology, clinical manifestations, diagnosis and management [4,7,8,9]. However, due to the relevant rarity of the disease, the lack of awareness of HPP among physicians and the absence of pathognomonic symptoms, especially in the mild adult form, there is still considerable delay in its diagnosis and management [10].

## 2. Pathophysiology

Biomineralization is a tightly regulated process by which the organisms produce mineralized tissues, such as bone and teeth [11]. It is dependent on the complex interplay between calcium, inorganic phosphate (Pi) and PPi, hormones, such as parathyroid hormone (PTH), vitamin D and Fibroblast Growth Factor 23 (FGF23), and enzymes, mainly TNAP. There are four genes that encode ALP, three tissue-specific, namely intestinal, placental and germ cell (*ALPI*, *ALPP*, *ALPG*, respectively), and the *ALPL* gene, expressed mainly in bone, liver, kidney and developing teeth, but also in the central nervous system, fibroblasts, endothelial cell and other cell types [1]. TNAP is an ectoenzyme, bound to the surface of plasma membranes via a glycosylphosphatidylinositol (GPI) anchor, that enables movement of the enzyme by enhancing membrane fluidity [12]. This GPI anchor can be cleaved by phospholipases found in plasma membranes, explaining how TNAP is released into the circulation. In bone, *TNAP* is highly expressed at the cell surface of osteoblasts and hypertrophic chondrocytes and forms homodimers to attain fully catalytic activity. The natural substrates of TNAP include PPi, PLP, and possibly di-phosphoryl lipopolysaccharide (LPS) and phosphorylated osteopontin (OPN) [9].

**Figure 1:**
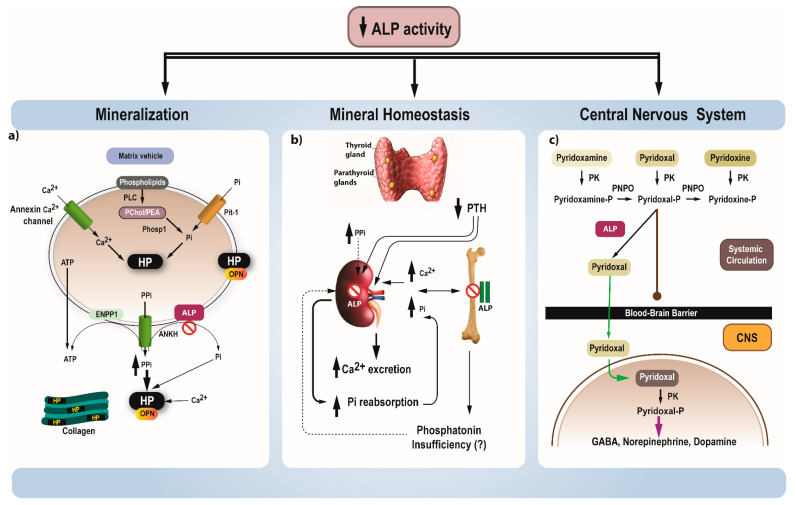
(**a**) Primary mineralization is initiated in the MV, which are buds of the osteoblast membrane. Pi produced within the MV by the action of PHOSPHO-1 on PChol and PEA derived from membrane phospholipids by the action of PLC and also transported intravesicularly by Pit-1, combines with calcium and forms hydroxyapatite crystals (Phase 1). Crystal growth results in the rupture of MV, then proceeds outside the MV, within the extracellular matrix and propagates on collagen fibrils (Phase 2). PPi, transported extravesicularly by ANKH and produced locally by the action of ENPP1 on ATP, is a strong inhibitor of mineralization. On the contrary TNAP promotes mineralization by hydrolyzing PPi, and by providing Pi. In HPP, phase 1 proceeds normally; however, elevated PPi levels due to deficient TNAP activity inhibits the mineralization of the skeletal matrix. Moreover, phosphorylated OPN, another inhibitor of mineralization, is also elevated in mouse models of HPP. (**b**) In severe cases with HPP, the blocked entry of calcium and Pi in the skeleton results in elevated calcium and Pi, appropriately followed by reduction of PTH levels and subsequent hypercalciuria. In mild cases, elevated Pi levels are observed, due to increased renal phosphate reabsorption. Possible explanations include direct role of TNAP in renal phosphate reabsorption, competition with excess PPi for the same transport mechanism and inappropriately normal or reduced levels of phosphatonins. (**c**) Of the vitamin B6 vitamers, only PLP can act as a cofactor for enzymes in the CNS. However, PLP must be dephoshoshorylated to PL in order to enter the cells and the blood brain barrier. TNAP accomplishes the dephosphorylation of PLP to PL, so it can pass the blood brain barrier. Within the cells PL is rephosphorylated by pyridoxal kinase to PLP, which acts as cofactor for GABA synthesis. Abbreviations: ALP, alkaline phosphatase, ANKH, ankylosis protein homolog; ATP, adenosine triphosphate; Ca, calcium; CNS, central nervous system; ENNPP1, ectonucleotide pyrophosphatase/phosphodiesterase; GABA, gamma-aminobutyric acid; HP, hydroxyapatite; HPP, hypophosphatasia; MV, matrix vesicles; OPN, osteopontin; PChol, phosphocholine; PEA, phoshoethanolamine; PK, pyridoxal kinase; PLC, phospholipase C; PNPO, pyridoxamine 5′-phosphate; Phosp-1, phosphate phosphatase 1; oxidase; Pi, Inorganic phosphate; Pit-1, phosphate transporter -1; PPi, pyrophosphate; PTH, parathyroid hormone; TNAP, tissue non-specific alkaline phosphatase.

Primary mineralization is initiated in the matrix vesicles (MV), which are buds of the osteoblast membrane (Figure 1a). Pi is produced within the MV, is transported intravesicularly by phosphate transporter -1 (Pit-1) and connects with calcium ions to form hydroxyapatite crystals (Phase 1). Crystal growth results in the rupture of MV, proceeds outside the MV within the extracellular matrix and propagates on collagen fibrils (Phase 2). TNAP promotes mineralization by hydrolyzing PPi, and by providing Pi. In HPP, phase 1 proceeds normally; however, elevated PPi levels due to deficient TNAP activity inhibits the skeletal matrix mineralization. Moreover, OPN, another inhibitor of mineralization, is also elevated in mouse models of HPP. In the growing skeleton, all these mechanisms lead to rickets, but to osteomalacia in adults [1,9]. In addition, deficient mineralization of acellular cementum results in premature loss of deciduous teeth.

Concerning mineral homeostasis, in severe cases, reduced incorporation of minerals in the skeleton results in elevated calcium and Pi, which, in turn, leads to reduced PTH levels and hypercalciuria (Figure 1b). However, in less severe cases, mineral homeostasis is normal, apart from the mild elevation of Pi levels and hypercalciuria. Hyperphosphatemia in particular, is mainly attributed to increased renal reabsorption of phosphate (elevated tubular maximum reabsorption rate of phosphate to glomerular filtration rate (TmP/GFR)) [12]. Although the underlying mechanism is unknown, possible explanations include direct role of ALP in renal phosphate reabsorption, competition with excess PPi for the same transport mechanism and inappropriately normal or reduced levels of phosphatonins. Indeed, a recent study in patients with pediatric HPP confirmed the presence of hyperphosphatemia, which was not correlated with the disease severity, the elevated TmP/GFR along with inappropriately normal FGF23 and secreted Frizzled Related Protein 4 (sFRP4) levels, and low levels of fibroblast growth factor 7 (FGF7) [12].

B6-dependent seizures represent a cardinal and unfavorable prognostic manifestation of perinatal and infantile HPP, considered to be due to vitamin B6 deficiency in the central nervous system (CNS) [13]. Of the vitamin B6 vitamers, namely pyridoxine, pyridoxal (PL) and pyridoxamine and their phosphorylated esters, only pyridoxal 5’ phosphate (PLP) can act as a cofactor for enzymes, such as γ-aminobutyric acid (GABA)-transaminase and glutamate decarboxylase in the CNS [14,15]. It should be highlighted that GABA is one of the main inhibitory neurotransmitters in the CNS. PLP must be dephoshoshorylated to PL, in order to enter into the cells and cross the blood brain barrier (Figure 1c). TNAP accomplishes the dephosphorylation of PLP to PL, so it can pass the blood brain barrier. Within the cells, PL is rephosphorylated by pyridoxal kinase to PLP, which acts as cofactor for GABA synthesis. Low levels of GABA have been reported in a mouse model of HPP [16], although several other mechanisms might be implicated.

## 3. Clinical Presentation

The first attempt to classify HPP was done by Donald Fraser in 1957 [2], after reviewing 35 cases with HPP. He described three major groups (infants, children and adults) based on the patients’ age when symptoms or lesions became apparent. Currently, HPP is classified into six forms [1,7], based on three factors: age of onset, severity and clinical manifestations.

### 3.1. Benign Prenatal HPP

This type refers to relatively mild skeletal abnormalities (e.g., short, undermineralized limbs) detected with antenatal ultrasonography in utero or at birth. They tend to improve spontaneously during late pregnancy or ex utero. Its natural history is variable, as it may progress from odontoHPP to the infantile severe form. Unfortunately, this cannot be predicted [17].

### 3.2. Perinatal HPP (OMIM 241500)

This is the most life-threatening type of HPP, inherited with the autosomal recessive trait and is rare (Canada 1/100,000, Europe 1/300,000, Japan 1/182,000) [18], with the exception of Mennonite families in Manitoba (Canada), in whom the prevalence is high (1/2500). Its manifestations are obvious by the end of pregnancy or at birth [19]. If untreated, death is almost always anticipated in utero or perinatally [20,21]. Serum ALP of the neonate is too low, or even undetectable [22].

Fetal ultrasonography reveals polyhydramnios, bowed and short long bones, low or absent skeletal mineralization and osteochondral spurs protruding from the forearms and legs, which are also evident on the skeletal survey postnatally and are accompanied by skin dimpling [23]. Profound skeletal hypomineralization also leads to caput membraneceum, with only central mineralization of calvarial bones, as well as hypoplastic thoracic cage with gracile ribs and fractures. The resulting pulmonary hypoplasia, along with tracheomalacia and profound muscular weakness leads to respiratory failure, the major cause of mortality in these patients. The radiological changes are characteristic and show bone hypomineralization, radiolucent areas at the extremities of long bones (“tongues”), slender and deformed bones, “moth-eaten” and cup-shaped metaphyses, and osteochondral spurs.

An important, adverse prognostic factor is the presence of pyridoxine-dependent seizures [24]. As mentioned above, TNAP dephosphorylates PLP to pyridoxal to cross the blood–brain barrier and regulate the synthesis of neuro-transmitters, e.g., the inhibitory neuro-transmitter gamma-aminobutyric acid. In severe cases of HPP this synthesis is unfeasible, thus leading to vitamin B6–dependent seizures. They initially respond to the administration of vitamin B6 but may become refractory at a later stage [25]. In cases in which sedatives and muscle relaxants mask the presence of epileptic activity, monitoring with an amplified electroencephalograph (EEG) may support prompt diagnosis of subclinical seizures, even from the first day of life [24].

Another potentially serious neurological complication is the presence of increased intracranial pressure, presenting with papilledema, headaches, and emesis, secondary to severe cranio-synostosis, which usually manifests before the age of five years. The lambdoid suture in particular tends to close early in HPP, thus restricting posterior fossa development and posing a risk for Chiari Type I malformation, i.e., herniation of cerebellar tonsils [26] and hydro-syringomyelia. When of late onset, it may induce few clinical signs. Thus, periodic monitoring of head circumference and fundoscopy are important at follow up.

In addition, the combination of high calcium levels in serum and urine explains the nephrocalcinosis seen in many perinatal cases, which may jeopardize renal function. Other non-specific signs and symptoms include irritability, fever, intracranial hemorrhage, bluish sclerae [27], myelophthisis (usually manifested as anemia) and predisposition to infections, because of low TNAP in leucocytes, which may impair their functionality [25,28].

### 3.3. Infantile HPP (OMIM 241500)

This is a moderately severe type of HPP, which usually follows an autosomal recessive type of inheritance. The affected newborns appear healthy at the time of birth, but later present with failure to thrive, poor feeding, muscular weakness, delay in achieving developmental milestones and signs resembling rickets, i.e., wide fontanelles and rachitic deformities [29].

Similar to the perinatal form, respiratory failure is a serious complication, as there is pulmonary hypoplasia, small thorax, gracile bones, recurrent fractures and tracheomalacia, accompanied by typical HPP radiological signs [30]. Moreover, there is often functional closure of cranial sutures, resulting in craniosynostosis and intracranial hypertension (papilledema on fundoscopy) in some cases. The presence of Wormian bones is also described in the literature [31]. Another complication, particularly at the age of 4–5 years [32], is nephro-calcinosis [29]. Hypercalcemia may, at least partly, explain the presence of irritability, poor feeding, anorexia, vomiting, polydipsia, polyuria, dehydration and constipation, sometimes observed in this type of HPP [33].

Poor prognosis is again predicted by the presence of respiratory failure (rib fractures, thoracic deformity, recurrent pneumonias) and/or pyridoxine-dependent seizures [20,25]. Periodic clinical and radiographic evaluation is important for prognostication, as ALP and PLP levels correlate with severity, but they do not represent reliable prognostic biomarkers. Mortality of this type, although lower than the perinatal type, remains high: untreated patients with infantile HPP have 50% mortality in the first year of life [20,21]. There are cases resembling infantile HPP, but with normal serum ALP activity, characterized as pseudohypophophatasia (pseudoHPP). Most cases of pseudoHPP are attributed to an inappropriate reference range of ALP or to its transient elevation.

### 3.4. Childhood HPP (OMIM 241510)

This type is further sub-divided into mild and severe. The mildly affected children are generally in good health, good physical function, and experience minor or no symptoms. They almost invariably report early tooth loss, and their radiographic skeletal changes are very subtle, e.g., low bone mass. On the other hand, severe childhood HPP can be challenging and heterogeneous. It is usually, but not always, inherited as an autosomal recessive trait and the patients, apart from the premature tooth loss, suffer from skeletal pain (leading to episodes of unexplained crying and refusal to walk) and muscle weakness, which is the cause of delayed walking, waddling gait, and difficulty in climbing stairs. Their skeletal deformities include pectus excavatum, which predisposes to restrictive pulmonary disease, misshapen skull due to underlying craniosynostosis, with a risk of raised intracranial pressure [34,35], scoliosis and deformed long bones due to slow-healing recurrent fractures. Furthermore, poor mineralization results in bowed legs or knock knees (genu varum or genu valgum, respectively) and swollen wrists (metaphyseal flaring). In female patients, the age at menarche is not influenced. As far as growth is concerned, its impairment is proportional to the degree of ALP levels and disease severity [19,36].

The key radiographic features of childhood HPP are evident early in the course of the disease: “tongues” of lucency projecting from the growth plates into the metaphyses, patchy osteosclerosis, osteopenia, wide, irregular physes and fractures, involving mainly the long bones, may be observed, whereas vertebral fractures are unusual. The orthopantogram reveals large pulp chambers and root canals (the so-called “shell teeth”). Moreover, bone mineral density (BMD), measured with dual-energy X-ray absorptiometry (DXA), may decline over time.

Presentation of severe HPP in adolescence is unusual, although mild cases can be diagnosed at this age. In late adolescence and young adulthood, there can be a relative improvement in skeletal symptoms (“honeymoon period” [37]), because of the growth plate fusion and the relatively smaller need for ALP function. However, these patients may frequently complain of muscle and joint stiffness and pain, as well as weakness of the legs and fatigue [38]. There are also HPP cases in adolescents mimicking chronic non-bacterial osteomyelitis or chronic recurrent multiple osteomyelitis, presenting with painful swellings of either the axial or the appendicular skeleton and areas of bone oedema in magnetic resonance imaging (MRI) [7,28,39]. In this case, malignancy should be excluded; total body MRI is a useful diagnostic tool. Finally, most of the teenagers suffering from HPP are relatively short compared to their peers, may also have dental problems, they may be dolichocephalic and their muscle bulk is often low, accompanied by joint laxity and typically genu valgum [18]. They may also report metatarsal stress fractures that are slow to heal [40].

Regarding prognosis, childhood HPP is considered a stable condition. However, dental and skeletal disorders, as well as joint involvement, reemerge during adulthood. Therefore, HPP diagnosed in childhood or adolescence should not be regarded as a benign condition and regular monitoring is recommended.

### 3.5. Adult HPP (OMIM 146300)

Adult HPP is usually milder than pediatric HPP, albeit very heterogeneous in terms of clinical manifestations [41]. At presentation, almost two-thirds of patients are symptomatic, primarily reporting musculoskeletal pain and/or fractures [41,42,43]. Musculoskeletal pain is a common complain (40–75% in different series), and may include foot, ankle, knee, thigh, hip pain, back pain, joint swelling/pain, or diffuse musculoskeletal pain [10,41,42,43,44]. A history of fractures is documented in 40–55% of patients with adult HPP, sometimes multiple (in about one-third of patients with fractures) [10,41,43]. The most common sites of fractures are the feet (e.g., metatarsal stress fractures) and femur/hip, but fractures can also occur in the wrist and the vertebrae or other bones. Fractures are usually slow to heal and may recur [1,42,43,44]. Implants for fracture fixation or joint replacement sometimes fail [23]. Interestingly, BMD can be low, normal, or high [45]. High lumbar spine BMD has been paradoxically associated with higher risk of fractures [45]. Dentition is also affected in adult HPP: early loss of adult teeth is a common feature (25–35%) [43,44]. However, some patients may have experienced a premature loss of their deciduous teeth with intact roots, long before the diagnosis of adult HPP [42,46].

A history of osteomalacia may also precede the diagnosis of HPP [1]. Pseudofractures, which are radiological hallmarks of osteomalacia, may occur (7%) [1,42], especially at the femur [41]. Due to fractures and osteomalacia, bowing deformities of long bones have been reported in some patients (about 15%) [41,42]. Radiographic calcific periarthritis or chondrocalcinosis, featuring deposition of hydroxyapatite around major joints or in cartilage, is observed in 10–25% of patients with adult HPP and may be symptomatic [41,42,43,47,48]. Pseudogout, featuring deposition of calcium pyrophosphate dihydrate, may be observed in about 5–15% of patients [41,42]. Ossification of ligaments (syndesmophytes) [49] and scoliosis have also been reported in some patients [50,51]. Other symptoms described in patients with adult HPP are headache (sometimes daily), chronic fatigue, unusual gait, as well as difficulty in sleeping, eating and/or swallowing [42,43,46].

It seems that adult HPP, especially its mild forms, may remain asymptomatic and, thus, largely underdiagnosed [52]; in this regard, most symptoms, e.g., musculoskeletal pain, are not specific for the disease, and are common in the general population. This sets an unclear borderline between adult HPP and normal phenotype and a diagnostic challenge, i.e., whether the symptoms of a patient heterozygous for an *ALPL* gene mutation may be attributed to this heterozygosity [4]. On the other hand, the more severe forms of adult HPP may compromise the quality of life, may require assistive devices for disability and/or home modifications and may be debilitating [1], mainly owing to the recurrent limb fracturing and the musculoskeletal and joint pain [53].

### 3.6. OdontoHPP (OMIM 146300)

This is the mildest and probably the most frequent form of HPP [19]. It is restricted to dental manifestations, without concomitant physical and/or radiological findings of the disease. There is painless, premature exfoliation of teeth (primary incisors) with the root intact (age < 5 years), without gingival inflammation, ulcerations, abscess, or history of trauma. There are cases that have been diagnosed with histology of the exfoliated teeth, in which the clinical suspicion was high, even in the absence of low ALP values [54]. As a general principle, the milder the HPP type, the less teeth are lost. In OdontoHPP, the average number of teeth lost before the age of five is four, compared, for instance, to the infantile HPP, in which the average number of lost teeth is nine [19].

It is crucial not to misinterpret as OdontoHPP other causes of early tooth loss, such as scurvy (vitamin C deficiency), severe periodontitis, hypophosphataemic rickets, dentinal dysplasia type I, neutropenias, leucocyte adhesion deficiency, histiocytosis and various syndromes, such as Papillon le Fevre, Chediak Higashi and Ehlers Danlos.

Primary dentition is more sensitive to low ALP function, and this is important, as premature tooth loss can affect nutrition and speech. The poorly mineralized cementum leads to decreased tooth root anchorage to the periodontal ligament. There are also large pulp chambers, thin dentin, and alveolar bone loss on the orthopantogram. However, these findings may improve, as the patient grows. The patients appear otherwise healthy, with no abnormalities on their X-rays. However, some of them may be slightly shorter compared to their peers. It is believed that OdontoHPP is not entirely benign, as it may evolve to adult HPP at a later stage, with chronic bone pain and stress fractures; however, this remains under investigation. It is important to note that patients with OdontoHPP are advised towards regular follow up for bone and other, non-skeletal complications, e.g., hypercalcemia, chronic pain, fatigue, low energy fractures and hypotonia.

Finally, carriers of HPP are defined as the subjects with low ALP activity or even defective *ALPL* allele(s), but no clinical manifestations of HPP. Investigation of family members and long-term follow-up is required to determine whether such carriers will develop manifestations of HPP.

## 4. Diagnosis and Counseling

### 4.1. Biochemical Tests

The cardinal biochemical feature of HPP is the persistently low serum ALP levels. However, since many different ALP assays are available, based on a series of detection strategies, such as colorimetry, fluorescence, and electrophoresis [55], the sensitivity and precision of the method used should be taken into consideration, when interpreting the results. The most widely used diagnostic method at the clinical level is colorimetric determination. The ALP activity is determined by measuring the rate of conversion of p-nitrophenyl phosphate (pNPP) to p-nitrophenol (pNP) in the presence of magnesium and zinc ions and of 2-amino-2-methyl-1-propanol (AMP) as a phosphate acceptor at pH 10.4. The rate of change in absorbance due to the formation of pNP is then measured bi-chromatically at 410/480 nm and this rate is a direct function of the ALP activity in the sample. Among the other isoforms bone (BALP) and liver TNAP isoforms are the most frequently present with a ratio of approximately 1:1 in the serum of healthy individuals [56]. As a marker of bone formation BALP is not only involved in physiological but also in pathological mineralization such as vascular calcification, or cancer [57]. However, it is not generally used, except for the cases of concomitant liver disease, where serum activity of ALP may be falsely within normal range. In the paediatric population, use of bone ALP is less popular, as it is significantly more elevated than liver ALP, because of bone modeling. The rise of chromatography techniques on the one hand have led to the development of high-performance chromatography methods for separation and quantification of ALP isozymes, while the increased use of immunoassays for serum BALP have helped overcome the previously used technique’s limitations (e.g., poor sensitivity and specificity) for total ALP measurement [57].

Most of the assays report their own reference range. However age- and sex-specific presentation of reference range is necessary to establish the diagnosis during growth [5], since fluctuations in ALP closely mirror bone growth. In specific ALP activity levels it is known to initially increase slightly during childhood, with the highest levels during the main growth period, while it declines after cessation of growth following puberty. Sex differences are also observed between 11 and 79 years, reflecting the unique sex-specific bone changes that occur throughout life [58]. Since ALP is a well-established biomarker for a series of diseases, other conditions that may interfere with ALP activity (Table 1A,B) should by carefully evaluated and excluded. Acute hypophosphatasemia has been recorded during periods of major trauma/surgery, multisystem failure, acute anemia, or acute caloric restriction, and is associated with profound illness or physiologic stress followed by increased short-term mortality [59]. On the other hand, adults with persistent hypophosphatasemia frequently harbor *ALPL* mutations and may fall within the spectrum of the adult form of hypophosphatasia. In cases like those, clinicians should be cautious in prescribing bisphosphonates when needed [60].

Nevertheless, a value less than 40 U/L for adults of both sexes should raise suspicion for HPP, in the absence of other diseases that may also present with lower concentrations of ALP (Table 1B).

Although it is well established that extremely low ALP levels correlate with clinical severity [61], HPP cases with ALP within the low normal range have also been described [7,61]. This may either reflect detection of a functionally less active protein by the assay or the presence of a concomitant disease or a condition expected to increase ALP levels, such as a recent fracture, severe osteomalacia or even pregnancy. In these cases, serial measurements of ALP may be necessary.

Of note, since the spectrum of diseases linking to abnormal ALP activity is considerably wide, measurement of the ALP substrates, such as urine concentration of PEA and PPi and serum PLP concentration, may also be considered. Increased urinary PEA measurement has been used for detecting HPP cases (urine amino acid assay), but it may be normal in affected individuals or elevated in other metabolic bone diseases and thus it is not considered a sensitive diagnostic marker.

Increased levels of PPi in the urine is also a sensitive biomarker in affected individuals and asymptomatic heterozygotes; however, urinary PPi is not specific for HPP, since increased levels may be found in a wide spectrum of diseases, such as acromegaly, uremia, pseudogout or osteoarthritis [62]. It is technically demanding and not widely available, hence its use is restricted to research settings.

Increased serum levels of PLP are also used for the diagnosis of HPP [63,64], although not widely available. Moreover, as the biologically active metabolite of vitamin B6, patients should be guided to refrain from the use of vitamin supplements for at least a week, as it may lead to false positive results. Parameters of calcium metabolism, including serum levels of calcium, Pi, PTH and vitamin D (both 25-hydroxy and 1,25-dihydroxy) are usually within normal range, and may help in the differential diagnosis from rickets. Hypercalciuria with or without hypercalcemia and hyperphosphatemia with hypophosphaturia may also be present, but their levels largely vary and are not helpful for diagnosis.

### 4.2. Genetics

HPP is most commonly caused by heterozygous or compound heterozygous mostly missense (about 74%) mutations at the *ALPL* gene [65]. The mode of inheritance refers to an autosomal dominant or recessive manner, with the most severe phenotypes being transmitted with the autosomal recessive trait, whereas the milder forms are transmitted with either the dominant or recessive trait [19]. In general, the severe phenotypes of HPP are usually caused by homozygous or compound heterozygous mutations [66] and manifest as a recessive disease. In cases of mild or moderate forms with autosomal dominant inheritance the underlying mechanisms involve either a dominant negative effect of a single heterozygous mutation, intronic mutations, or mutations in the regulatory sequence [65,67]. In European patients it has been shown that 13.4% of the HPP-affected chromosome alleles have a dominant negative effect [3].

Apart from the prenatal benign form, every other phenotype demonstrates significant genetic heterogeneity with 3–5 distinct genotypes, reflecting the high variability of the clinical spectrum [4]. In a recently published cohort of 424 unrelated HPP patients consisting of 166 heterozygotes and 258 homozygotes or compound heterozygotes, it has been demonstrated that HPP forms based on their genetic characteristics and their prevalence can be subdivided into three distinguishable subtypes: the severe, moderate, and mild adult subtypes [4]. The severe HPP forms are mostly caused by homozygosity or compound heterozygosity for severe variants; moderate HPP forms are produced by dominant negative effect of missense variants; while mild adult HPP, that are usually characterized by unspecific signs, are probably related to haploinsufficiency mechanisms. Regarding the new mechanism of dominance, although not yet clearly identified, it has been hypothesized that an interaction between TNAP and other factors that can trigger insufficiency may be implicated [4].

The genetic diagnosis is based on the identification of the *ALPL* mutation and is critical for the diagnosis of prenatal HPP, although it is not considered to be a prerequisite for the diagnosis of the other types of HPP. Molecular testing approaches include serial single-gene testing, in which a sequence analysis of *ALPL* gene is followed by a gene-targeted deletion/duplication analysis, if only one or no pathogenic variant is found, or a multigene panel strategy, which may include, except for *ALPL*, other genes of interest. Sequence analysis may detect small intragenic deletions/insertions and missense, nonsense, or splice site variants that can be pathogenic, but also benign or of uncertain significance. Methods used may include quantitative polymerase chain reaction (PCR), long-range PCR, multiplex ligation-dependent probe amplification (MLPA), or a gene-targeted microarray designed to detect single-exon deletions or duplications [4,68]. Mutations in the non-coding regions of the gene that affect the functionality of the protein are also reported, although not currently considered in clinical practice. In these cases, a more comprehensive genomic testing, including exome and genome sequencing, may also be used, according to which whole-gene deletions or duplications may be detected. In cases where a single-gene panel fail to confirm a diagnosis in an individual highly suspicious for HPP, other genes that can regulate TNSALP activity such as RUNX2, that is critical for differentiation and bone formation, should also be considered [69]

In severe (perinatal and infantile) HPP, two *ALPL* pathogenic variants are identified in approximately 95% of individuals of European ancestry, while in other forms one or even two *ALPL* pathogenic variants may be detected [68,70]. In general, in more moderate HPP forms about 50% have two *ALPL* pathogenic variants (compound heterozygote or homozygote), while 40–45% present with one identified pathogenic variant. In milder forms of the disease, usually only one ALPL pathogenic variant is detected [4]. No data on detection rate of gene-targeted deletion/duplication analysis are currently available, whereas a few HPP cases with deletions have been reported [71].

## 5. Management

Since HPP is a rare disease, it is important that the patients be managed in experienced referral centers or in close coordination with their referral centers [9,23]. This practice has reduced the time of diagnostic uncertainty and inappropriate care of the affected individuals [23]. It is also important that a multidisciplinary team is available, consisting of orthopedic surgeons, pediatricians, dentists, endocrinologists, neurologists, physiatrists, pulmonologists, and nutritionists, who are expected to co-operate so as to optimize the management of HPP patients.

### 5.1. Conventional Management

Conventional management in HPP is symptomatic, i.e., driven by the major symptoms and signs of each affected individual. In babies with HPP, respiratory support with mechanical ventilation may be required in a neonatal intensive care unit [72]. In cases of seizures, anti-epileptics are administered together with pyridoxine [25]. For symptomatic craniosynostosis, a craniotomy may be required performed by a neurosurgeon [1]. In case that hypercalcemia is evident, dietary calcium should be decreased; hydration, loop diuretics and/or glucocorticosteroids may be used, as necessary [1]. Vitamin D deficiency should be restored, with concomitant avoidance of excessive doses of vitamin D, in order to prevent excessive calcium and phosphate absorption [73]. Anti-reflux treatment may also be needed in patients with severe gastroesophageal reflux and failure to thrive. In the most severe cases, nutrition through gastric or jejunal tubes may be temporarily required for sufficient weight gain [73]. Analgesic medications, e.g., non-steroidal anti-inflammatory drugs, are needed to treat pain; however, the renal function should be carefully monitored, to avoid renal toxicity [74]. Dental care is of paramount importance. In children, early education about rigorous oral hygiene is essential to avoid or retard periodontitis [73]. Removable prostheses may be required in young children, until the permanent teeth arise. Dental implants may be used in adults, when necessary [73]. Fractures and pseudofractures should be managed appropriately; when delay fracture healing is evident, prolonged use of casting or stabilization with intramedullary rods is necessary [73].

Regarding bone-targeted therapies, teriparatide (TPTD), a recombinant parathyroid hormone (1–34) approved for the treatment of osteoporosis [75], seems to improve bone pain and benefit the healing of fracture and pseudofractures in adults with HPP [76,77]. TPTD has also been administered “off label” to some adults with HPP, targeting to upregulate the synthesis of more TNAP in osteoblasts [76,77,78]. According to this rationale, the presence of one normal *ALPL* allele is required, as TPTD treatment to upregulate the transcription of the intact *ALPL* gene, i.e., this may work only in heterozygosity [1,79]. Nonetheless, TPTD has helped some, but not all [80], patients with HPP [76]. An important consideration is that TPTD has been approved for two years for osteoporosis [75], which renders a time deadlock in the management of patients with HPP, because it is expected that *ALPL* expression will decrease after TPTD discontinuation, even in patients having initially responded to this treatment. It should, however, be highlighted that TPTD treatment may be prolonged for patients with osteoporosis and persisting or recurring fractures (https://dailymed.nlm.nih.gov/dailymed/fda/fdaDrugXsl.cfm?setid=aae667c5-381f-4f92-93df-2ed6158d07b0&type=display) (27 November 2021), which is the case in some patients with HPP. Less frequent than daily TPTD administration has been also used in adult HPP, so as to prolong its use for more than two instances, with favorable results [79].

Other authors have used bone marrow transplantation in infants or growth hormone therapy in children with HPP with reportedly satisfactory results [65]. However, until more data are available, their use should be carefully considered on an individual basis. BPS804, an anti-sclerostin monoclonal antibody, has also been administered for four months in eight patients with adult HPP leading to increased bone formation and bone mineral density [81]. However, studies in osteoporosis showed a rapid decline in bone mineral density after the discontinuation of romosozumab, another anti-sclerostin monoclonal antibody [82], which may result in undesirable consequences in patients with HPP; thus more data are needed on the use of romosozumab in HPP, especially when it is discontinued.

Antiresorptive medications, e.g., bisphosphonates, denosumab, are not considered to be suitable management for patients with HPP, because they further suppress bone turnover and ALP activity, thus aggravating underlying osteo-malacia [83,84]. Atypical femoral fractures have also been described after bisphosphonate treatment in patients with adult HPP [83,85].

Historical attempts to manage HPP are elsewhere described in detail [1]. They include, but are not limited to, cortisone administration and intravenous infusions of ALP purified by various sources, e.g., plasma of patients with Paget’s bone disease or placenta. They largely failed, thus possibly implying the need for upregulating *ALPL* in the skeleton [1].

### 5.2. Enzyme Replacement Therapy for HPP

#### 5.2.1. Pediatric Population

In 2015, asfotase alfa (Strensiq^®^, Alexion Pharmaceuticals Inc, Boston, MA, USA), a recombinant, bone-targeted, human TNAP was approved for the treatment of bone manifestations of HPP in Japan (all types) and for perinatal, infantile, and severe childhood-onset HPP in Canada, the United States and the European Union. It constitutes a long-term enzyme replacement therapy (ERT). The drug contains the catalytic homodimeric soluble TNAP domain, the human immunoglobulin G1 Fc region (which aims to prolong the circulating half-life) and deca-aspartate residues for hydroxyapatite targeting [86,87]. For mild childhood and odonto-HPP, careful follow-up is usually sufficient, as the skeletal profile is not severely affected and there is not enough evidence that dental phenotype can be reversed with treatment [88,89]. With regards to benign prenatal HPP, caution must be exerted in the differential diagnosis to perinatal HPP. A “watch and wait” approach seems reasonable in this scenario, as there is a chance of spontaneous clinical improvement [18].

Asfotase alfa approval was granted after the conduction of global clinical studies across the paediatric age range, which showed improvement of bone mineralization, respiratory function, long-term survival, and a favorable safety profile (Table 2) in patients suffering from moderate to severe skeletal HPP manifestations. More specifically, Whyte et al. performed a multicenter, multinational, open-label, phase 2 study, which included 37 patients with perinatal or infantile HPP and 48 historical controls, matched for age and disease severity. Asfotase alfa was given subcutaneously either at 2 mg/kg, three times/wk. or at 1 mg/kg, six times/wk. During the five-year follow up period, the need for mechanical respiratory support was found to be lower in the treatment arm and there was marked improvement in radiographic severity scores, i.e., improved mineralization, which allowed better stabilization of the chest and more optimal respiratory mechanics. Another important outcome of this study was survival; 95% vs. 42% at age 1 year and 84% vs. 27% at 5 years, for treated patients vs. historical controls, respectively [20].

Asfotase alfa therapy was also associated with gradual improvement of metaphyseal sclerosis and normalization of the epiphyses after 24 weeks of treatment [29]. In 2019, an extension study was published by the same group and described the 7-year follow-up of 11 infants and young children diagnosed with perinatal or infantile HPP. The authors reached the same conclusions, as it was confirmed that there was not only improvement in the skeletal manifestations and respiratory function (no need for respiratory support after the fourth year of ERT), but also improvement in growth (normalized median weight Z-scores from the third year of treatment and improvement in median length after six months on ERT), cognitive (tested by Bayley scales of infant and toddler development, 3rd ed (Bayley-III)) and motor function. Apparently, the benefits of treatment were present for up to seven years of therapy [89]. In terms of side effects, the most frequent were pyrexia and upper respiratory tract infection (73% of the patients); these were mild-to-moderate in severity and were managed accordingly [89].

A small group of adolescents with pediatric-onset HPP have also been studied, with regards to asfotase alfa efficacy and safety, following the effects of asfotase alfa in a group of six adolescents and seven adults with HPP [92]. The control group consisted of six untreated patients. In the treated group, asfotase alfa dose escalated gradually to 6 mg/kg/week. After six months of treatment, TNAP activity was higher and its substrates (i.e., PLP and PPi) decreased within the reference range. Apart from the improved biochemical profile, there was also significant improvement in the six-minute walk test, reflecting better mobility and functionality. There was no death or serious adverse effects during follow-up. Musculoskeletal pain also seems to be relieved by ERT, according to another review. Apparently, ERT improves weakness and thus performance of everyday tasks and this is an important outcome for the patients [93]. It is advised to monitor progression of scoliosis in teenagers with HPP on asfotase alfa, given that there is a case report describing worsening of scoliosis at one year of ERT, perhaps due to an acceleration of skeletal growth [51]. In female adolescents on ERT, appropriate contraceptive measures should be discussed, as the drug safety during pregnancy and lactation has not been established.

According to current recommendations, asfotase alfa should be administered subcutaneously at a total dose of 6 mg/kg/wk., which is the maximum recommended dose (half-life: 5 days), given either three days/wk. (2 mg/kg/d) or six days/wk. (1 mg/kg/d). In exceptional circumstances, when treatment response is suboptimal, this dose can be increased to a maximum of 9/mg/kg/wk. in perinatal, infantile or childhood-onset HPP, after the appropriate exclusion all other possible causes of treatment failure, e.g., nutritional issues, presence of scoliosis or craniosynostosis, detection of anti-drug antibodies or non-compliance with treatment [94]. Treatment decisions regarding ERT warrant expertise on HPP and its complications, so prompt referral to an expert center is advised. Treatment failure has been reported in very severe cases, even after administrating high ERT doses, apparently due to the coexistence of extremely severe lung hypoplasia [95]. In these severely affected infants, long periods of critical care are necessary (weeks to months) before skeletal mineralization and respiratory function improve [40].

Follow-up of patients on asfotase alfa should be regular and detailed, tailored to the patient’s phenotype and clinical needs and, as mentioned above, should be multidisciplinary. It includes weight measurement every three months in order to adjust treatment dose. If dose is suboptimal or discontinued, weakness reappears, followed by radiographic deterioration [34,96]. Skeletal radiographs, combined with functional assessment (respiratory function, pain, mobility, and quality of life evaluation), depending on patient’s age, are also necessary [97], as well as regular growth assessment and biochemical tests. It is also important that the patients avoid obesity, to prevent its comorbidities, but also to be more functional, in terms of mobility. Guidelines for the monitoring of patients with HPP who are on asfotase alfa are presented in more detail elsewhere [94].

According to another study of 20 patients (including three adults) with HPP on ERT, a useful monitoring marker for assessing treatment compliance and efficacy is serum PLP/PL ratio, which changes during treatment, before the skeletal effects of asfotase alfa are evident. The authors claim that a PLP/PL ratio < 4 implies good compliance and efficacy of treatment and allows proper titration and optimization of the dose, especially when the high cost of asfotase alfa is considered [86,98]. Calcium metabolism should also be monitored during treatment. While on ERT, bone mineralization improves, therefore, calcium intake requirements are increased (“hungry bone syndrome”). Adequate calcium supply is essential, along with regular measuring of calcium in serum and urine, as well as PTH levels [94]. Of note, increased PTH levels were shown to be in parallel with skeletal healing [29].

Regarding safety, all available studies on asfotase alfa show that it is well tolerated. Very common side effects are the local reactions at the injection sites. These are usually mild-to-moderate and include transient erythema with pruritus, followed by chronic purplish discoloration and local wrinkling, sometimes with lipohypertrophy, the specific mechanism of which remains largely unknown. To avoid these reactions, rotation of injection sites is strongly recommended [94]. Moreover, when the drug volume exceeds 1 mL, it should be given with multiple injections at the same time. Pyrexia is also a very common adverse event, along with headache. In a Japanese cohort of 13 patients, a serious side effect, possibly related to treatment, was the presence of severe hypocalcaemia, accompanied with seizures in one patient with the perinatal form. There were also three patients that presented with hypercalcaemia and required a low-calcium diet. No episode of anaphylaxis was noted [91]. In another cohort of 11 children with perinatal or infantile HPP, serious adverse events attributed to ERT were: a post-injection reaction (skin erythema, abdominal pain, dizziness, headache, rigors); an episode of possibly drug-induced, chronic hepatitis (albeit there was history of coadministration with montelucast, the discontinuation of which led to clinical improvement); and a case with severe progression of craniosynostosis accompanied by conductive deafness [89]. However, it should be noted that craniosynostosis is a common manifestation of treatment-naïve patients with the same HPP type [99] and that ERT has made surgical correction of HPP-related craniosynostosis safer and more effective [26]. In addition, ectopic calcifications (e.g., conjunctiva, cornea) have been reported. However, they are not considered clinically significant (i.e., do not affect vision) and it is unclear whether they are the results of the disease or the treatment [34]. According to the summary of product characteristics (SPC) of the drug, hypersensitivity reactions in the form of anaphylaxis have been rarely described and mandate discontinuation of ERT; if re-challenge of ERT is decided, this should be performed under medical supervision and premedication, e.g., proper administration of anti-histaminic medications.

Anti-asfotase antibodies have been detected in some patients, but their clinical relevance, e.g., loss of efficacy, hypersensitivity or need for dose change, are reportedly minimal. However, Hofmann et al. showed, in a cohort of 69 HPP paediatric patients with perinatal or infantile HPP, that the radiographic non-responders had more severe disease at baseline and more frequently neutralizing antibodies at the last assessment [90]. In line, according to a case report [100], a patient on ERT for 2.5 years developed anti-asfotase alfa antibodies that were associated with loss of treatment efficacy. To manage this, the patient discontinued ERT for a month, received immunotolerance induction therapy and ERT efficacy was restored within six months of its re-initiation. For the time being, access to anti-asfotase alfa antibody testing is possible only through the Global HPP Registry, which collects relevant data, thus targeting to the evaluation of the impact of the antidrug antibodies on the clinical course. This Registry also collects data on the effectiveness, safety and tolerability of asfotase alfa and also on the natural history and clinical burden of HPP worldwide [42].

#### 5.2.2. Adult Population

The long-term efficacy and safety of asfotase alfa in adults and adolescence with paediatric onset HPP has also been published [92]. In this 5-year phase 2 study, 13 adults and 6 adolescents, after a 6-month primary treatment period, in which they were randomized to receive asfotase alfa at doses 2.1 mg/kg/wk. or 3.5 mg/kg/wk. or no treatment, all received asfotase alfa at a dose of 6 mg/kg/wk. Evaluation of efficacy included the change in plasma PLP and PPi, BMD, bone mineralization (transiliac bone biopsy), walking ability (6-min walk test) and several other measures, such as pain, muscle strength and functionality. PLP and PPi concentrations were reduced in all patients, and efficacy was maintained for the 5 years of treatment. Concerning BMD, either increase or decrease in z-scores at the LS, total hip and whole-body were observed; however, at all-time points, z-scores remained within normal range. Mean osteoid volume decreased with therapy, while it increased in the control group. Mineralization lag time improved (−580%), even with the low dose during the initial, primary treatment period. Walking ability improved, use of assistive ambulatory devices was reduced, while pain was reportedly decreased in most patients. Similar results concerning physical function and health-related quality of life among adults with HPP were reported by another group [101] Treatment was generally safe; efficacy was maintained, and no major adverse effects were reported during the 5-year period. Nine patients developed asymptomatic conjunctival calcifications, an effect that remains unclear as to whether it is related to treatment with asfotase alfa or to the natural history of the disease. Similarly favorable results were reported in several case reports in adults with HPP [102,103,104,105,106]. In addition, Seefried et al. [107] reported the 24-month effect of asfotase alfa on bone turnover markers and BMD in 21 adults with paediatric-onset HPP. Serum osteocalcin, procollagen-1 N-terminal peptide and PTH increased at 3 and 6 months and returned to baseline at 12 months, while tartrate-resistant acid phosphatase 5b increased at 3 months and returned to baseline thereafter. Calcium, phosphate, eGFR and FGF23 levels did not change, while BMD increased. The authors interpreted these changes as indicative of the improvement in bone remodeling following treatment. Based on these findings, any effect of asfotase alfa on bone markers seems to be transient; however, further studies are needed to validate these findings.

Delayed fracture healing is a common severe skeletal manifestation of HPP, with variable responses to conservative measures, including off-label use of TPTD. Thus, treatment with asfotase alfa might be considered a rational indication. Successful treatment with asfotase alfa has been reported in two adult patients with HPP [108], a 41-year-old woman with a tibial and bilateral femoral pseudofractures and a 61-year-old man with bilateral femur fractures. Remarkably, complete fracture healing was observed after 11 and 16 months of ERT, even at fractures remaining unhealed (nonunion) for 3 and 17 years, respectively. In addition, Stürznickel et al. [109], reported continuously improvement in BV/TV values after asfotase therapy in three adults with HPP and nonunion, using three-dimensional evaluation of cone-beam computed tomography images, thus the successful consolidation and mineralization of osteoid was implied.

Currently, ERT has been approved for the treatment of bone manifestations in patients of all ages with paediatric-onset HPP. Treatment with asfotase alfa substantially improves clinical manifestations of the disease and most importantly improves survival and quality of life. At least in severe cases, treatment should be continued for long-term after the completion of growth since discontinuation of ERT may probably lead to clinical deterioration. For adults with HPP, ERT may be considered in the presence of the following [110,111]: (1) Major low-trauma fractures or pseudofractures; (2) delayed or incomplete fracture healing or fracture nonunion; (3) intractable musculoskeletal pain or chondrocalcinosis requiring or unresponsive to opioids; (4) disabling functional impairment with impaired gait and mobility assessed by validated measures. Nonetheless, it should be noted that asfotase alpha is currently approved for the treatment of adult HPP only in Japan. The high cost of the medication probably renders its wider use unaffordable for the health systems and patients, thus resulting in a therapeutic deadlock [79]. On the other hand, enzyme replacement therapy may, at least theoretically, limit the number of hospital visits and medical interventions (e.g., major orthopaedic surgeries) required for severely affected patients, thus avoiding considerable healthcare costs, which are mostly related to disease severity and complications.

### 5.3. Counseling

The phenotypic spectrum of HPP is widely variable and depends on the age of onset, the mode of inheritance or the type of gene deletion or duplication. The alleles composing the so far described genotypes are classified according to functional tests; however, not all variants have been tested [112] and, therefore, the use of genetic testing to predict the phenotype is not always accurate.

Genetic counseling should also consider the presence of signs not associated to HPP, the family history and genotyping of both parents and siblings.

Most importantly, information must be given during pregnancy focusing on the genotyping of prenatal HPP cases in an attempt to differentiate between lethal and benign HPP cases and also to detect cases who will benefit from prompt enzyme replacement therapy during the perinatal period [113].

When neither parent of a proband with an autosomal dominant condition has the pathogenic variant identified, then the pathogenic variant is likely to be de novo, but in these cases other diagnoses (Table 1B) or alternate paternity or maternity (e.g., with assisted reproduction) should be considered.

## 6. Closing Remarks

HPP is a rare inherited systemic metabolic disease, with a wide spectrum of clinical manifestations and severity. However, its “rarity” is questionable, given the low awareness of the disease, the little attention paid by clinicians to low ALP levels and the mild non-specific phenotype observed in several more common metabolic bone diseases.

Concerning the manifestations of the disease, more data are needed about the natural history of HPP, especially after the completion of growth, and the long-term consequences and survival, especially of the milder cases, if left untreated. The above mentioned HPP registry is expected to provide useful data in this regard. As far as diagnosis is concerned, it is important to develop a simple diagnostic protocol for the detection of HPP cases and to identify useful markers, either genetic and/or biochemical, to accomplish reliable prognostication of disease severity in cases and “carriers” of HPP. Although ERT with asfotase alfa is straightforward in severe cases of paediatric onset, more data are needed regarding the effectiveness of treatment, especially in adult HPP, its optimal duration, and the required dose of ERT in milder cases with HPP. Finally, careful cost-benefit analysis of the use of enzyme replacement therapy across all age ranges in different healthcare settings is eagerly required, in an effort to provide all patients with the best possible standards of care.

## Figures and Tables

**Table 1:** Conditions associated with abnormal (A. elevated, B. reduced) serum levels of ALP.

(**A**)	
**Conditions Leading to High ALP Levels**	
Cholestasis (extrahepatic)	**Non-hepatic causes**
Malignant obstruction (pancreas, gallbladder, bile duct)	Healing fractures
Primary sclerosing cholangitis with extrahepatic bile duct stricture	Osteomalacia
Infection (e.g., AIDS cholangiopathy, Ascaris lumbricoides)	Paget’s disease of bone
Cholestasis (intrahepatic)	Osteogenic sarcoma, bone metastasis
Primary biliary cholangitis	Hyperparathyroidism
Primary sclerosing cholangitis	Hyperthyroidism
Total parenteral nutrition	Myeloid metaplasia
Infiltrative diseases	Peritonitis
Alcohol-associated hepatitis	Subacute thyroiditis
Sickle cell disease (hepatic crisis)	Gastric ulcer
Metastatic carcinoma to the liver	Osteosarcoma
**Hepatic causes**	**Extrahepatic tumors**
Hepatitis (viral, non-alcoholic, alcoholic, drug-induced)	Lung, gastric, head and neck
Liver cirrhosis	Renal cell
Infiltrative diseases of the liver	Ovarian, uterine cancers
(**B**)	
**Conditions leading to low ALP levels**	
**Endocrine conditions**	Vitamin C deficiency
Cushing syndrome	Zinc or magnesium deficiency
Hypothyroidism	Vitamin D intoxication
**Metabolic bone diseases**	**Others**
Osteogenesis imperfecta, type II	Wilson disease
*Cleidocranial dysplasia*	Celiac disease
*Hypophosphatasia*	Cardiac bypass surgery
*Mseleni joint disease*	Hemochromatosis
*Benign familial hypophosphatasemia*	Radioactive heavy metal intoxication
Adynamic bone disease	Improper specimen collection
**Hematological conditions**	**Drugs**
Massive transfusion	Clofibrate
Pernicious or profound anemia	Bisphosphonates
Multiple myeloma	Denosumab
**Diet related**	Tamoxifene
Milk-alkali syndrome	Glucocorticoids
Starvation	Chemotherapy

Conditions associated with persistently low ALP are shown in *italics*.

**Table 2:** Clinical studies of asfotase-alfa in paediatric patients.

Reference	No of Patients	Age Range (Median)	Bone Mineralization	Respiratory Outcome	Survival Rate	Safety	Comments
Whyte MP et al., NEJM 2012 [29]	11	2 wks–3 y	↑RGI-C: +2 (wk 24), +2.3 (wk 48)	6/9 on air at wk 48	82% alive at year 1	SIRs common 3 SAEs:respiratory distress, craniosynostosis, deafness	AA dose: 3–9 mg/kg/wk
Whyte MP et al., Lancet Diabetes Endocrinol 2019 [89]	9	3–158 wk (30 wk)	↑RGI-C: at least +2 at year 6	No respiratory support at year 4	100%	PyrexiaURTI:73%, 3 SAEs: severe chronic hepatitis, moderate ISR, severe craniosynostosis and deafness	Extension study (7 y follow up), AA dose: 3–9 mg/kg/wk
Whyte MP et al., JCEM 2016 [20]	37	0–71 mo (9 mo)	↑RGI-C: at least +2 of those weaned from ventilation	75% weaning ventilation	95% at 1 y, 84% at 5 y, 76% of the ventilated patients	NA	Median AA treatment duration: 2.7 y (6 mg/kg/wk)
Whyte MP et al., JCI Insight 2016 [34]	12	6 y–12 y (8.6 y)	↑RGI-C: +2.2 at year 5	NA	100%	No SAES, SIRs common (erythema 85%, lipohypertrophy 62%)	5 y follow-up, AA dose: 6 or 9 mg/kg/wk
Hofmann CE et al., JCEM 2019 [90]	69	0.02–72 mo (16 mo)	↑RGI-C: +2 at year 1	11/24 patients no longer needing support	13% died 80% alive at year 6	Pyrexia: 68%, SIRs common anaphylaxis: 1/69 drug hypersensitivity: 1/69	Non-responders: 28% (more severe HPP, ↑rate of AA-Abs)
Kitaoka T et al., Clin Endocrinol (Oxf) 2017 [91]	13	0–34 y (3 mo)	↓RSS: –5.4 ± 2.7 at 24 wk	37.5% off ventilator 2/13 weaning ventilation	100% at 24 wk	ISRs: 53.8% 1 patient with hypo-calcemic seizures	AA dose: 6 mg/kg/wk

Abbreviations: AA, asfotase alfa; SIRs; AA Abs, Asfotase alfa neutralizing Antibodies; NA, Non-Applicable; RCI-C, Radiographic Global Impression of Change; RSS, Rickets Severity Score; Site Injection Reactions; SAEs, Severe Adverse Events.

## Data Availability

Not applicable.
